# Nanoparticle transport pathways into tumors

**DOI:** 10.1007/s11051-018-4273-8

**Published:** 2018-06-21

**Authors:** S. M. Moghimi, D. Simberg

**Affiliations:** 1 School of Pharmacy, The Faculty of Medical Sciences, Newcastle University, King George VI Building, Newcastle upon Tyne, NE1 7RU UK; 2 Division of Stratified Medicine, Biomarkers & Therapeutics, Institute of Cellular Medicine, Newcastle University, Framlington Place, Newcastle upon Tyne, NE2 4HH UK; 3 Translational Bio-Nanosciences Laboratory and Colorado Center for Nanomedicine and Nanosafety, The Skaggs School of Pharmacy and Pharmaceutical Sciences, Department of Pharmaceutical Sciences, University of Colorado Denver, Anschutz Medical Campus, 12850 East Montview Blvd, Aurora, CO 80045 USA

**Keywords:** Interendothelial transport, Nanomedicine, Nanoparticle, Solid tumors, Transendothelial transport, Nanoparticle accumulation in tumors

## Abstract

Two transport pathways (interendothelial and transendothelial routes) have long been proposed for entry of nanoparticles from the blood circulation into solid tumors. We examine and discuss available evidence supporting interendothelial and transendothelial transport processes and suggest new avenues for re-evaluating these pathways. Understanding of integrative mechanisms controlling nanoparticle extravasation into tumors is important for improving engineering and performance of anti-cancer nanopharmaceuticals.

## Introduction

The enhanced permeability and retention effect (EPR) has become an equivocal concept in the targeting and development of anti-cancer nanomedicines. This concept has been recently scrutinized due to disappointing therapeutic efficacy and limited clinical success with anti-cancer nanomedicines compared with excellent results in small animal xenograft models (Anchordoquy et al. 2017; Bjornholm et al. 2017; Moghimi and Farhangrazi 2014; Nel et al. 2017; Park 2013). The complexities and heterogeneity associated with the EPR effect have now been confirmed in high-resolution imaging studies in dogs with primary tumors, but these attempts have yet to resolve nanomedicine transport pathways into tumors as well as their variability within the same tumor mass (Hansen et al. 2015). Two transport pathways for translocation of nanoparticles from the blood circulation into the solid tumors (interendothelial and transendothelial pathways) have been proposed (Bjornholm et al. 2017; Chauhan et al. 2012; Cabral et al. 2011; Dvorak and Feng 2001; Fang and Nakamura 2011; Gerlowski and Jain 1986; Hobbs et al. 1998; Liu et al. 2017; Matsumura and Maeda 1986; Taurin et al. 2012; Yuan et al. 1995). The interendothelial pathway considers nanomedicine extravasation from the blood into tumor interstitium through the pores or open fenestrations in the tumor blood vessels, whereas the latter relies on transcytosis by tumor endothelial cells. What are the evidence supporting these pathways?

## Transendothelial route

Limited morphological evidence support operation of transendothelial route in some tumors, and biomarkers supporting transendothelial transport are becoming available (Dvorak and Feng 2001; Thurston et al. 1998; Liu et al. 2017). For instance, one study reported the presence of more fluorescently labeled cationic liposomes in the endosomes and multivesicular bodies of the tumor-associated endothelial cells than in adjacent normal vessels in a β cell carcinoma model spontaneously developed in the pancreas of RIP-Tag transgenic mice (Thurston et al. 1998). More recently, ultrastructural and functional studies in an orthotopic tumor model in mice strongly supported dominant involvement of a transcytosis pathway involving a vesicular network of similar appearance to vesiculo-vascular organelles (Dvorak and Feng 2001) (and triggered by neurophilin-1, which is expressed on tumor blood vessels) in nanoparticle transport into solid tumors (Liu et al. 2017). The vascular neurophilin-1 expression apparently regulates the extent of nanoparticle translocation into tumors (Pang et al. 2014) and may serve as a biomarker for predicting the tumor response to nanopharmaceutical delivery (Liu et al. 2017).

Mouse tumor xenografts often have high vascular density with notable contributions from cutaneous vascular network, since tumor cells are usually inoculated subcutaneously (Anchordoquy et al. 2017; Bjornholm et al. 2017; Moghimi and Farhangrazi 2014; Nel et al. 2017; Park 2013; Taurin et al. 2012). Recently, we demonstrated rapid skin deposition of intravenously injected liposomes regardless of their lipid composition and circulatory half-lives (Griffin et al. 2017). This process presumably involved liposome translocation across the skin capillaries, with an active role for endothelial cells in uptake and transcytosis. Therefore, it is plausible that in xenografts, the rich cutaneous vascular network may increasingly contribute to transvascular transport of nanoparticles through active particle uptake and transcytosis by endothelial cells, and account for the major difference seen in particle accumulation kinetics between human tumors and mouse xenograft models.

## Interendothelial route

It has been difficult to trace nanoparticle transport through interendothelial fenestrations, and this may have been due to technological deficiencies (such as poor resolution of intravital microscopy in observing nanoparticle dynamics) as well as many pathophysiological factors such as size heterogeneity seen in tumor vessel fenestrae (100–1200 nm), pore frequency/density, other architectural abnormalities, and stochastic intratumoral pressure (Fang and Nakamura 2011; Hobbs et al. 1998). The indirect evidence in favor of interendothelial transport pathway, however, has been based on the observation that large-sized particles (> 250 nm) can extravasate into various tumors (Yuan et al. 1995; Lee et al. 2013; Key et al. 2015), but the size range of some of these particles still fits the dimensions of endocytic compartments. Nevertheless, in Kaposi sarcoma, where tumor vessels are highly “leaky,” an electron microscopy study did not find direct evidence of nanoparticle migration across the pores (Huang et al. 1993). Others have also questioned whether pore-dependent mechanism of extravasation is truly operative, and have argued that extravasation may not solely be controlled by nanoparticle size (Smith et al. 2012). Similar to tumor blood vessels, sinusoidal capillaries in the liver also contain pores, allowing particle extravasation into the hepatic parenchyma (Wisse et al. 1985). Studies evaluating particle extravasation into hepatic parenchyma have identified a number of parameters controlling interendothelial transport, which may also be relevant to tumors. For instance, liposome size was shown not to be a decisive factor regulating passage across the pores (Daemen et al. 1997; Romero et al. 1999; Scherphof and Kamps 2001). On the other hand, liposome rigidity and phospholipid headgroup structure were playing modulatory roles. Liposomes, depending on their extent of deformability, could pass through the narrow fenestrations by forced “extrusion” involving red blood cells (Daemen et al. 1997; Romero et al. 1999; Scherphof and Kamps 2001). This is analogous with a process that regulates fluid exchange between the sinusoid and the space of Disse (“endothelial massaging”) (Wisse et al. 1985). However, not all fluid liposome types were “squeezable” through the fenestrations. This led to the hypothesis that for successful extravasation some interactions between liposomes and endothelium are necessary to retain vesicles (and hence the role of phospholipid headgroup structure) and drive extrusion by fast flowing erythrocytes (Daemen et al. 1997; Romero et al. 1999; Scherphof and Kamps 2001). On this basis, particle extravasation through open fenestrations in the tumor blood vessels may not only depend on nanoparticle dimensions and the extent of particle deformability/rigidity, but also on weak/moderate interaction with endothelium to enable “retention.” If particle retention on tumor endothelium is a prerequisite for successful erythrocyte-mediated forced extravasation, then the efficiency of endothelial massaging will not only be dependent on local fluid dynamics, such as the apparent blood viscosity, blood flow rates in the tumor capillaries, and intratumoral pressure gradient, but also on particle shape (as this may modulate the extent of particle rolling on vessel walls), capillary diameter (which may control erythrocyte stretching and deformability, and hence collision rates with nanoparticles), and surface modification. Nevertheless, some surface strategies (e.g., ligand and polymer grafting) that promote particle retention on endothelium might also increase particle transport via transcytosis. The modulatory role of nanoparticle acquired blood protein corona (Moghimi et al. 2012) on endothelial retention and transcytosis also remains to be elucidated.

There have been many other recent studies mapping particle extravasation mechanisms into tumors, and yet none has resolved transport pathways satisfactorily. For instance, one study has proposed vascular bursts as an alternative explanation, but this study could not exclude the role of transendothelial transport as the explanation for bursts (Matsumoto et al. 2016). Mathematical models of size-dependent nanoparticle extravasation have also been built, showing that reducing the sizes of pores through vessel normalization decreases the interstitial fluid pressure in tumors and allows small nanoparticles to enter more rapidly (Chauhan et al. 2012). Collectively, these models a priori are based on pore hypothesis and could not differentiate between interendothelial and transendothelial transport pathways (Sykes et al. 2014).

## Conclusions

The widespread use of xenograft models and complexities surrounding tumor endothelial cell biology and vessel architecture (e.g., vascular structural disorganization, irregular branching, and uneven distribution density) has contributed to ambiguity in transport pathways across tumor endothelium. Furthermore, the interaction between nanoparticles and vessel walls (and subsequent extravasation processes) might be controlled by the perfusing concentration of nanoparticles (temporal dimension) as well as capillary architecture, and integrated mechanical and fluid dynamic factors that modulate local blood cell (e.g., erythrocyte) stretching. Here, nanoparticle physicochemical properties such as size, shape, deformability, and surface characteristics all play important roles in establishing nanoparticle impaction with blood cells, contact with endothelium, and rolling on vessel walls. Recent developments in nanoparticle engineering include precision control of particle shape, mechanical stiffness, and surface properties (Moghimi et al. 2012). These technological developments could offer an important tool for revisiting transport pathways in tumors systematically. In addition to these, some nanoparticles can physically adsorb to erythrocytes, and when injected intravenously exhibit prolonged circulation times in the blood (Pan et al. 2018; Wibroe et al. 2017). Accordingly, erythrocyte-bound particles (of different morphologies and dimensions) may also prove useful at least in assessing the endothelial massaging phenomenon in nanoparticle extravasation. Understanding the mechanisms of extravasation of nanoparticulate systems is key to improving pharmacokinetics and tumor targeting of anti-cancer nanomedicines.
